# Dr. Bouillaud on Fibrinous Coagula in the Heart

**Published:** 1839-08-03

**Authors:** 


### BIBLIOGRAPHICAL NOTICE.

### DR. BOUILLAUD ON FIBRINOUS COAGULA IN THE HEART.

[From " L'Experience."]

In one of the preceding numbers of the Examiner, some remarks were contained in a lecture by one of the editors, upon the inflammation of the heart and arteries, which often complicates pneumonia. This inflammation is, in most cases, accompanied by the formation of fibrinous coagula in the heart, which appear, at times, to be the effect, and, at other times, the cause of the inflammatory action. As soon as the coagula begin to form, the dyspnœa of the patient increases very rapidly; and in the majority of cases, death ensues much sooner and more frequently than it would do if the disease of the lungs were not complicated with the cardiac disorder. The proportion of cases of pneumonia which terminate fatally would be much reduced, were it not for this complication; indeed, the disease would very rarely prove fatal, unless the patient were enfeebled by advanced age or a previous disorder. It, therefore, is extremely important to arrive at a correct diagnosis of their cause, and to learn the best means of treatment.

The diagnosis, it will be seen by the memoir of Dr. Bouillaud, is, in general, very easy, with the assistance of the physical signs; and even without their aid, the collateral circumstances are, in general, sufficient to render the case tolerably clear. Our experience coincides with that of the Professor of La Charité, and we have ascertained that the lesion is quite as frequent in this country as in France; we are, therefore, much pleased to be able to furnish our readers with a translation of the deductions which he has drawn from his very extended observation. We translate them from a late number of "L'Expérience."

As to the treatment: when the coagula are formed, the most effectual remedies are cupping over the region of the heart, followed in many cases by dry cups over the posterior parts of the thorax; mustard foot-baths and sinapisms, to excite the circulation of the skin, and the administration of diffusible stimulants, which act most directly upon the heart. Of these latter remedies, the most useful are ether, and Hoffman's anodyne; the carbonate of ammonia and assafœtida may often be combined with them, to the great advantage of the patient. The object, in short, is two-fold: to relieve, in some measure, the heart of the accumulation of blood, and, at the same time, to keep up the powers of this organ. Dr. Bouillaud recommends large bleedings,—this is in accordance with his views of the treatment of pneumonia; but we entirely dissent from them in reference to the coagula of the heart. After the coagula have begun to form, large bleedings are out of place, and tend directly to enfeeble the action of the heart; when this organ is, to a certain extent, relieved, they may again become appropriate,—but, in general, large abstractions of blood are well suited to the treatment of the early stages of inflammatory pneumonia, but not to the more advanced periods of the disorder. In this respect, we regret that the theoretical views of Dr. Bouillaud have been carried farther than is consistent with prudence or impartial observation.

*Anatomical character, and manner of formation of the Sanguineous Fibrinous Coagula, observed in the preceding cases.*

'I shall not (says M. Bouillaud) mention all the details of the various anatomical appearances presented in the preceding cases, but will merely state, that in all instances the coagula presented the character of those which Corvisart and other pathologists have considered as indicating that they were formed during life. Corvisart states, that this form of coagula are to be recognised by their pale colour, by their density, by their fibrous or fibrinous formation, and finally by their strong adherence to some part of the cardiac cavities. I cannot, however, suppose that the large masses of coagula which we observed in the heart and large vessels were entirely formed many days before death. A portion of these coagula undoubtedly was so, but the remainder was formed in the last moment of life and after death.

To a certain extent, the formation of the coagula occurring after death resembles that of blood drawn from a vein during life; thus, in the same manner that crassamentum of the blood from a vein is covered with a thick fibrinous buffy coat, &c., so the thick coagulated portions of blood formed at the moment or soon after death, present in various degrees the same plastic, white, fibrous coat. These coagula may, therefore, very properly, be designated by the term of sizy or buffy, in the same manner as we speak of the sizy buffy coat of blood to which we have compared it.

Whatever, may be the value of this analogy, which might be extended still further, it is very certain that in fourteen preceding cases the coagula were formed under the influence of pure febrile inflammation. If it be demanded that I should show the *modus operandi* of this influence, I would reply that it is not more known than that which causes the formation of coagula in inflamed vessels. My object now, is less to explain how these fibrinous coagula are formed, than to prove the reality of their existence. The explanation will eventually be given. It unquestionably presents a rich and magnificent subject of research to those physiologists who are capable of properly applying chemistry and physics in the investigation of vital phenomena, and to the causes producing these phenomena. Such researches on the fibrinous coagula which are so frequently formed in the heart and great vessels under the influence of high febrile inflammation would elucidate at the same time, not only the important phenomenon of the formation of the sizy coat, but also the phenomenon not less curious of the glutinosity of the crassamentum (as I have described it in the *Clinique Medicale et le journal hebdomadaire*,) and would illustrate also the pathological conditions in which the phenomenon occur. It being very well known that the blood has a strong tendency to coagulate in the *idiopathic* inflammations of the heart and blood vessels, we could, *àpriori*, announce that the same phenomena would occur in all cases of febrile reaction, which accompany great inflammation, pleura-pneumonia, for example. In fact, what are these reactions? Do they not arise directly from the inflammation of the sanguiferous system, or, are they not consequent upon the sympathetic irritation of that system? This is so true, that instead of simple sympathetic irritation, it is occasionally seen, as we have often observed in our patients, that true inflammation of the internal membrane exists. It should not, however, be forgotten, that in pleuro-pneumonia, besides the reaction *called sympathetic*, which affects the blood, the cardiac and vascular system as in all other severe inflammations, the proximity of the inflamed organs to the heart and the large vessels of the chest, is a condition which contributes greatly to the extension and propagation of the inflammatory action. Very numerous facts have clearly proven to us the tendency which inflammation has to attack organs contiguous to the part primatively affected: it is thus, for example, in pleurisy, and pleuro-pneumonia of the left side; the inflammation extends to the heart and its enveloping membranes, often also to the spleen and its fibro-serous capsule; in pleurisy of the right-side, the inflammation extending to the liver and its investing membrane, causes phlegmasia of various grades of action. These facts are *coincidences* in disease of the highest importance, and upon which we have greatly insisted for many years past.

In conclusion, in all cases of death from pure (légitime) acute pneumonia which have occurred in our practice, we have found in the heart and great vessels fibrinous coagula, evidently formed in part before death. We have also met with similar coagula in cases were death had resulted from other pure inflammatory diseases. Whatever may be the precise epoch when the coagula in question are formed, we do not hesitate to present the following as a *law of pathological anatomy; that in all subjects dying in the second or third stage of pure acute, well characterized pneumonia, these fibrinous coagula will be found.*

Should any *well observed* cases be presented in which such fibrinous coagula should not exist, it would merely recall this maxim, that " the exception confirms the rule, instead of destroying it." I say *cases well observed.* In fact, should all those cases where the observers have neglected noting the presence and appearance of the fibrinous coagula be considered as contrary to the law we have laid down, it would lead to a strange error. In order to find these coagula it is necessary to examine, with care, the heart, the great vessels, and the blood which they contain. Now, in how many cases is this neglected. At this moment I have before me the reports of a dozen cases of fatal pleuro-pneumonia collected in a clinical service. Well, in not one of these reports is the least mention made of the state of the heart, or of the great vessels, or of the blood which they contained.

I am aware that this conclusion differs from the opinion of my learned colleague, Dr. Bouvier, who, in reference to the coagula found by him in a case of pleuro-pneumonia, says: "Should these coagula be considered as arising from a particular state of the blood in pleuro-pneumonia?" I would further observe that these coagula are not found in numerous cases of acute pleurisy and pneumonia, as I was enabled to verify two days after the death of the patient above alluded to, by examining a female, aged eighty-five years, who had died of the same disease. I am also aware that my conclusion is not in entire conformity with the letter and spirit of the doctrine of M. Laennec, viz., "that it is not among the young and plethoric individuals, full of life and whose orgasm is eminently inflammatory, that the polypous coagula are suddenly (tout à coup) formed." Time will manifest which of the above opinions is correct, and will show which is the sincere expression of the exact observation of facts.

*Diagnosis of Fibrinous Coagula,—importance of this diagnosis in the prognosis and treatment.*

In twelve out of the fourteen cases which we have reported, we have *diagnosticated* positively, or only announced as probable, the existence of the fibrinous coagula. Now the diagnosis was based on the signs which we have given in "le traite clinique des maladies du cœur, et dans la clinique médicale de l'hôpital de la charité." But to speak merely of those signs derived from auscultation of the heart, and from the pulse, I would remark, that they were all present in a greater or less degree in every case of the present series. The patients to whom I allude presented the following physical signs:

*First patient.*—Action of heart tumultuous, sounds dull, indistinct, scarcely perceptible; pulse irregular, intermittent.

*Second patient.*—Sounds of heart confused, indistinct (etouffés); pulse small volume contrasting with strength of patient, the violence and extent of pneumonia.

*Third patient.*—Sounds of heart obscure, indistinct; pulse small, hobbling.

*Fourth patient.*—Strong bellows sound, synchronous with the pulse in the upper third of sternum.

*Fifth patient.*—(Heart not sufficiently auscultated.)

*Sixth patient.*—Sounds of heart almost extinct; diminution in the volume of the pulse.

*Seventh patient.*—Sounds of heart obscure, very indistinct (etouffé); pulse very small.

*Eighth patient.*—Sounds of heart dull, obscure, rough, indistinct; pulse thread-like.

*Ninth patient.*—Heart not properly examined.

*Tenth patient.*—No pulse in left arm; pulse in right arm very small and rapid, composed of thread-like vibrations, irregular, unequal, intermittent; impulse of heart tumultuous, irregular, unequal, intermittent, and so rapid that it was impossible to count the strokes; sounds of the heart dull, laboured, quick, with *rasp* murmur.*

*Eleventh patient.*—Sounds of heart obscure, very indistinct.

*Twelfth patient.*—Sounds of heart obscure.

*Thirteenth patient.*—Bellows sound in the first time; purring murmur in the præcordial region.†

*Fourteenth patient.*—Sounds of heart almost obliterated, and replaced by an indistinct, dull and confused murmur.

It must be borne in mind that to form a perfectly satisfactory diagnosis of the fibrinous coagula in the heart and great vessels, either at its commencement or in its more or less advanced stages, that not only the above signs should be called into requisition, but that the other circumstances which we have indicated in part, at least in the first article of these researches should also be attended to.

Those physicians who may think that the diagnosis of the existence of the fibrinous coagula of the heart and great vessels is an object of mere curiosity, commit a great mistake. It is not so; in fact, in exact clinical medicine, it is of high importance for the prognosis, that we should be able to know, whether a pleuro-pneumonia is complicated or not by the presence of such coagula. This complication renders the prognosis highly serious, for when these coagula are abundant, they constitute a great obstacle to the blood through the cavities of the heart and large vessels.

Unquestionably, it is not useless as regards sound practice, resulting from enlightened therapuetic views, to be able to discriminate whether the complication exists or not in a case of pleuro-pneumonia. It is a generally received opinion, that in this disease the pulse is full, large, and developed; it is, however, by no means rare to meet with a pulse oppressed, of small volume, which is in strong contrast with the extent and intensity of the pneumonia, and very frequently with the force of the impulse of the heart. Under such circumstances, most practitioners recommend that blood should not be abstracted.

* Previous to the pneumonia, this patient had been attacked with a cardiac affection, induration of the mitral valve and contraction of the orifice auriculo-ventriculaire; this disease was not then accompanied by the signs above mentioned.

† The subject of observation, anteriorly to the pneumonia, had been affected by a fibro-cartilaginous induration of the aortic valves which rendered us undecided as to the true cause of the bellows sound in his case.

They consider the smallness of the pulse as an infallible sign of radical debility, of pure adynamia, and they embrace this opinion more readily as with the smallness of the pulse there is associated great feebleness, and extreme prostration of muscular strength, and a tendency more or less marked to syncope. In a large majority of these cases, however, all these signs have no direct essential connection with the dynamic state of the system, or with a radical weakness of what is called *vital force*, but is owing rather to the presence of coagula beginning to form or already formed in the heart; under such circumstances sanguine emissions must not be abandoned; instant recourse should be had to them, in conformity to the rules for blood-letting laid down in several of our works, and more especially in our *Clinique Medicale.* How many cases might we not report here, which would demonstrate that, used with a wise and prudent boldness, blood-letting has speedily restored to the pulse its usual volume, its normal freedom, caused the swoonings to cease, increased the strength instead of prostrating it, and finally has saved the patient? I do not exaggerate when I say, that I possess the reports of more than forty cases of pleuro-pneumonia exhibiting all the symptoms and proving all the facts just mentioned.

